# Scientific iconoclasm and active imagination: synthetic cells as techno-scientific mandalas

**DOI:** 10.1186/s40504-018-0075-0

**Published:** 2018-05-14

**Authors:** Hub Zwart

**Affiliations:** Department of Philosophy and Science Studies (Chair), Faculty of Science, Institute for Science in Society (ISIS), Radboud University Nijmegen, Heyendaalseweg 135, 6525 AJ Nijmegen, the Netherlands

## Abstract

Metaphors allow us to come to terms with abstract and complex information, by comparing it to something which is structured, familiar and concrete. Although modern science is “iconoclastic”, as Gaston Bachelard phrases it (i.e. bent on replacing living entities by symbolic data: e.g. biochemical and mathematical symbols and codes), scientists are at the same time prolific producers of metaphoric images themselves. Synthetic biology is an outstanding example of a technoscientific discourse replete with metaphors, including textual metaphors such as the “Morse code” of life, the “barcode” of life and the “book” of life. This paper focuses on a different type of metaphor, however, namely on the archetypal metaphor of the mandala as a symbol of restored unity and wholeness. Notably, mandala images emerge in textual materials (papers, posters, PowerPoints, etc.) related to one of the new “frontiers” of contemporary technoscience, namely the building of a synthetic cell: a laboratory artefact that functions like a cell and is even able to replicate itself. The mandala symbol suggests that, after living systems have been successfully reduced to the elementary building blocks and barcodes of life, the time has now come to put these fragments together again. We can only claim to understand life, synthetic cell experts argue, if we are able to technically reproduce a fully functioning cell. This holistic turn towards the cell as a meaningful whole (a total work of techno-art) also requires convergence at the “subject pole”: the building of a synthetic cell as a practice of the self, representing a turn towards integration, of multiple perspectives and various forms of expertise.

## Introduction: science as performative ontology

As McLeod and Nerlich (2017) point out in their editorial for this thematic series, metaphors are fundamental tools for thinking about and interacting with the world, and this also applies to metaphors emerging in synthetic biology discourse (Balmer & Herreman 2009; Hellsten & Nerlich 2011; Boldt 2016). Although (or rather, because) this type of discourse is fairly technical, metaphors help us to come to terms with what is inherently abstract and complex, notably by comparing it to something which is familiar and concrete (Balmer & Herreman 2009; Hellsten & Nerlich 2011; Boldt 2016; McLeod & Nerlich 2017). Synthetic biology represents a turning point, moreover, for whereas twentieth-century life sciences research was bent on uncovering the elementary particles of life (a trend which culminated in the massive production of genomics sequencing data, Zwart 2012), synthetic biology conveys a more holistic orientation, by focussing on convergence (on the living cell as a systemic whole), but also by bringing multiple research fields together in the context of an overarching research program, a “Gesamtwissenschaft” (Zwart 2018). Furthermore, as Tomita (2001) and others have claimed, the final objective and major challenge of contemporary synthetic biology is the production of a functioning and self-replicating synthetic entity: a synthetic cell (also known as artificial cell or protocell).

McLeod and Nerlich likewise consider the creation of synthetic cells as one of the main currents of synthetic biological research (2017, p. 4). As an embedded philosopher, the author of this paper is involved in a synthetic cell project named BaSyC, an acronym which stands for *Building a Synthetic Cell*.^1^ In the *Scientific Summary* of this project it is claimed that building a synthetic cell represents “one of the grand *intellectual* challenges of the 21st century”, raising scientific and technological, but also *philosophical* and *ethical* questions (my italics). The BaSyC project explicitly aims to address a “big” scientific and ontological question: “What is life?”, and the grounding idea is that we can only really understand life when we are able to technologically reproduce it in vitro, in the form of a fully functional, self-replicating cell. Thus, BaSyC is not only a technoscientific endeavour, but also represents a case study in performative ontology.

Precisely because synthetic cell projects are devoted to a hypothetical object (to something which is inexistent and fictitious as yet), imaginative metaphors are bound to play a decisive role. And even the key signifier “cell”, one of the primordial terms of modern scientific biology as such, is definitely a metaphor, introduced by Robert Hooke in his science classic *Micrographia* (Hooke 1665). The cells which he spotted in cork through his microscope reminded him of the rooms of monks in a monastery. The “cell” signifier thus began its impressive discursive career as an image that was consciously *transferred* from the realm of human culture (monastic architecture) into the realm of organic nature (“metaphor” comes from μεταφέρειν and means: “to transfer”, “to carry across” in Greek). Subsequently, this metaphor has been transferred back again from the biological realm into a number of technical domains, such as for instance electronics, giving rise to terms like “cell phone”, which is short-hand for “*cellular* electronic network” (MacDonald 1979). Thus, the history of the cell-concept already points to a fascinating paradox. On the one hand, as French philosopher of science Gaston Bachelard argued, modern science is decidedly “iconoclastic” (Bachelard 1947, p. 77; Bachelard, 1953, p. 122), i.e. bent on replacing images and imaginative explanations by tested, rational concepts and quantitative relationships (measurement, equations, mathematical symbols and the like). At the same time, scientists are prolific producers of powerful metaphorical images themselves (from *cells* and the *double helix* in biology up to *black holes* and the *Big Bang* in astrophysics). In other words, science is both a destroyer and a producer of metaphors. This explains why, notwithstanding the iconoclastic tendency at work in scientific research, synthetic biology discourse is replete with metaphors.

This article explores the observation that, in scientific efforts to visualise the synthetic cell endeavour, one particular metaphor seems especially striking, namely the mandala metaphor: the tendency to represent synthetic cells with the help of mandala-like images (Zwart 2018). Synthetic cell visualisations often take the form of circular-quadratic diagrams, with a nucleus and a spherical membrane, suggesting recovered wholeness, as Carl Gustav Jung (1950/1959) argued. According to Jung, by suggesting unity and completion, mandalas compensate for disruptive, fragmented and chaotic features of the actual situation, and may even provide a visual aid or roadmap for researchers towards convergence, i.e. towards the development of a more comprehensive, holistic view.

This paper explores the role of mandalas (as metaphors of wholeness) in synthetic biology from a psychoanalytical (more specifically: Jungian-Bachelardian) perspective. First, I will point out that the tension (already indicated above) between scientific *iconoclasm* and scientific *iconogenesis*, between destroying and producing images, goes back to a distinction already made by Aristotle, but later taken up by Jung (1911/1968), namely between *rational* and *imaginative* thinking. In fact, Delbrück (1971), one of the founding fathers of molecular life sciences research, claimed that contemporary life sciences convey an Aristotelean view of life. According to Delbrück, the idea that the *visual form* (phenotype) of living beings is determined by a *logical program* (genotype) that *realises* itself in living organisms, is decidedly Aristotelean. This view of life was further elaborated by Erwin Schrödinger (1944/1967), and eventually resulted in synthetic biology: the effort to build artificial systems that *mimic* biological cells, based on our understanding of the *logos* of life (represented by the metaphor of the *code*). Subsequently, I will focus on the mandala metaphor as an archetype of recovered wholeness and completion, not only in the sense that a synthetic cell would be the final completion of a long and eventful journey of discovery (beginning with the disclosure of the molecular structure of DNA in 1953), but also in the sense that this type of research may actually be regarded as a practice of “individuation” on the part of the scientific subject. After fragmentation and specialisation, time has now come to put the pieces back together again, not only at the object pole of the knowledge process (moving from the elementary particles of life to the cell as a recovered whole), but also at the subject pole (shifting from specialisation to convergence and transdisciplinary research, even fostering the science-humanities dialogue). At the same time, the mandala as a symbol of unity and wholeness may easily obfuscate instances of disruption, tension and conflict emerging in actual laboratory life.

## Aristotle on the *form* and *formula* of living beings

Aristotle (1980, 192b) defined nature as the non-artificial: that which is not produced by us. And yet, in principle, nature is intelligible for human beings. According to Aristotle, human beings are *logical* animals (ζῷον λόγον ἔχον) and therefore able to discern the intelligible “logic” (λόγος) pervading living nature. This also applies to living beings. Aristotle regards them as composites of form and matter, so that human beings (as logical animals) are able to discern the *form* (εἶδος) or *formula* (λόγος) that constitutes a living being (Aristotle 1986, 402a, 415b). Indeed, all living beings are realisations or actualisations (ἐντελέχεια, 412a) of their formula or plan (λόγος, 412b, 415b). Therefore, Max Delbrück credited Aristotle with having anticipated “the principle implied in DNA” (1973, p. 55). Whereas the *form* (εἶδος) of living beings corresponds with their phenotype (their visual appearance), their plan or *formula* (λόγος) corresponds with their genome: the molecular program that *realises* itself in a particular organism. In short, the visual form or *Gestalt* (εἶδος) of a living being is the realisation of an inherent program (λόγος).

According to Aristotle, this distinction between visual form (εἶδος) and logical program (λόγος) is also reflected in our *understanding* of living entities. On the one hand, Aristotle sees human understanding as a continuation of visual perception. Whereas our eyes perceive living entities as compounds of form and matter, our understanding is focussed on the form (εἶδος) stripped of matter, so that thinking is a more abstract version of sense perception. In other words, whereas perception focusses on external things (πράγματα), the soul reflects on their inner images (φαντάσματα). Human understanding may also focus, however, on the *formula* or plan (λόγος) of living beings. Seen from this perspective, Aristotle argues, human understanding is comparable to reading letters (γραμματείον, 430a). Thinking in the sense of considering formula is comparable to mentally reading or writing a text. Thus, Aristotle introduces a distinction between two types of thinking, namely thinking as considering *images* (φαντάσματα) versus thinking as considering *characters* (γράμματα). And whereas the former focusses on the visual “form” (εἶδος), the latter is rather oriented towards discerning the “formula” or plan (λόγος) that is realised in the actual organism.

Aristotle explains the difference with the help of an example. If we see a beacon, we initially recognise it as fire: an entity with a particular, recognisable, visual form; until it begins to move, for then we realise that it actually is a signal signifying something (for instance: the approach of a vessel). Thus, Aristotle already makes a distinction between fire as a gestalt (*image*) and fire as a *symbol*, i.e. an element in an alphabet of signals, bearing a human signature. In contemporary philosophy, notably in the work of psychoanalyst Jacques Lacan, this evolved into the distinction between the *imaginary* (focussed on images or φαντάσματα) and the *symbolic* (focussed on symbols or signifiers: on γράμματα).

Carl Gustav Jung (1911/1968) likewise distinguished these two types of thinking. While imaginative thinking builds on mental images (Aristotle’s φαντάσματα), rational thinking is directed by concepts and arguments: by logic. And whereas imaginative thinking is associative and free-floating, rational thinking operates on the basis of linguistic, logical and mathematical principles (and is therefore more demanding and exhausting, mentally speaking). Finally, whereas imaginative thinking is the oldest form of thinking (more attuned to the spontaneous functioning of the human mind), rational thinking is a more recent acquisition, historically speaking. Important intellectual developments, ranging from the invention of reading and writing via scholasticism up to modern science have contributed to its current dominance. But logical thinking has never completely replaced or erased imaginative thinking, so that the tension between both types of thinking (between the imaginary and the symbolic) continues to exist, *even in contemporary technoscience* as we have seen.

This distinction is also reflected in the history of biology as such, where we discern a shift of focus from the visual shape (εἶδος or appearance, which is the subject matter of morphology) to the symbolic dimension (i.e. the plan, the program, the code, the λόγος of life). Whereas in the eighteenth and nineteenth centuries scholarly inquiries were first and foremost oriented on exploring the visual, morphological form or structure of an organism, on the *gestalt* of living entities, as exemplified by the work of Goethe (1817/1824), contemporary biosciences rather focus on the codes and programs of living systems: on the symbolic or λόγος dimension.^2^

According to Gaston Bachelard, this is quite in line with the “iconoclastic” tendency of modern laboratory science (Bachelard 1947, p. 77; Bachelard 1953, p. 122). Bachelard was a Jung-adept who developed a psychoanalytical diagnostics of the natural sciences, focussing on chemistry, physics and biology. On the one hand he emphasised modern science’s aversion against images and the imaginary, notably in the sense that scientific research challenges our narcissistic self-images (the idea of human beings as something exceptional and unique) and tends to disrupt imaginary (e.g. mythological and religious) worldviews. Although iconoclasm began as a religious concept, Bachelard argues that it became a distinctive feature of modern science as well (Bachelard 1947, p. 77; Bachelard 1953, p. 122), most notably of the performative, experimental branches of research, whose objective is to understand nature or natural entities, not by *letting nature be* (as in artistic meditation or poetic exaltation), but by actively *transforming* natural entities into something symbolical (e.g. bio-chemical molecules and processes, captured in formula, symbols, equations and the like) with the help of laboratory equipment (technicity). Thus, the visible gestalt of a tree, for instance, is made intelligible for logical animals by reducing it to chemical letters and symbols (CO_2_, H_2_O, C_6_H_10_O_5_, etc.). Via symbolisation and literation, living beings are literally *obliterated* (Zwart 2016): they disappear from view; their visual form (εἶδος) becomes eclipsed, while the focus of attention shifts to their plan or formula (λόγος). The program of the life sciences of the twentieth century can be summarised as a shift of focus from *form* (εἶδος) to *formula* (λόγος), and from the organism as a visible and tangible *gestalt* (εἶδος) to life as a legible *code* (λόγος). At the same time, Bachelard emphasised that the imaginary (the imaginative style of thinking) cannot be repressed once and for all and will continue to resurge, even in scientific discourse. Scientists are prolific producers of images themselves, as we have seen, and prone to employ powerful metaphors to elucidate their abstruse ideas.

## The code of life

The understanding of living beings as *realisations* of a molecular *program* was also the grounding concept of quantum physicist Erwin Schrödinger in his science classic *What is Life?* (Schrödinger, 1944/1967). From a physics point of view, Schrödinger argues, life seems something highly exceptional, aberrational even, compared to abiotic nature. Nature as such is under the sway of the entropy principle: the process of inevitable and relentless decay. Anything that is well-ordered and complex is transient and bound to return to dust. How can something as complex, sophisticated and intricate as a living organism emerge, maintain and even reproduce itself in an entropic environment? Life, for Schrödinger, is “negative entropy”, i.e. the remarkable ability to withstand the pervasive, disruptive natural tendency towards pulverisation. How is this possible?

For Schrödinger, life is possible because of the program or code (Aristotle’s λόγος): the “genom” (spelled without an *e* by Schrödinger), an “aperiodic crystal” which carries a molecular “Morse code” (as Schrödinger metaphorically phrases it) that allows living cells to keep themselves *in shape* and even to replicate themselves. This code consists of strands of letter-like elements or characters (Aristotle’s γράμματα) which *realise* themselves in living organisms. Inspired by Schrödinger’s vision (Zwart 2013), Watson and Crick were indeed able to uncover the basic molecular logic of this code: the γράμματα (A, C, G and T) which constitute the nucleotide alphabet. Living cells are realisations of this program. And it is because of this logical program that human beings, as *logical* animals (ζῷον λόγον ἔχον) are able to read the intelligible λόγος pervading living nature, with the help of high-tech sequencing equipment.

In the final decades of the twentieth century, the research program (unleashed by Schrödinger’s book) culminated in the *Human Genome Project* (HGP). And now that the genomes of thousands of species have been sequenced, analysed, stored and published, another dialectical turn sets in, namely the shift from analysis to re-synthesis, from reading to rewriting (Zwart 2012), from reductionism to holism and reconstruction (Moya et al. 2008), in short: from genomics to synthetic biology as the new “frontier of science” (Ceccarelli 2013).

In order to understand how cells operate, modern science initially aimed to analyse them, by disclosing the basic molecular components, the strands of symbols that orchestrate the functioning and self-replication of cells. But how can we know that this process of analysis is really completed? The idea is that the only way to ascertain that we have managed to understand how living cells function, is to *realise* their program ourselves (in vitro), by producing a synthetic cell (Russel et al. 2012; Carrera & Covert 2015; Van den Belt 2009). The development of a synthetic cell (also known as artificial cell or protocell) is expected to “illuminate the perennial question ‘What is life?’” (Rasmussen et al. 2017). And as Murtas (2009) argues, the construction of artificial cells has now become a realistic option. Synthetic biology enables scientists to construct synthetic cells in a truly *bottom-up* fashion, moreover, by synthesising “all the essential biochemical mechanisms to yield a functionally and structurally understood self-replicating biosystem” (Murtas 2009, p. 1292). Such a project will produce “a deep understanding” of all cellular mechanisms and processes. Complex living cells produced in vivo (as products of evolution) raise the question whether all this complexity is really necessary for life, or whether cellular life can also be realised in human laboratories, involving much smaller degrees of complexity (p. 1293). Porcar et al. (2010) likewise argue that advances in DNA synthesis and a better understanding of regulatory processes make the goal of constructing an artificial cell a realistic possibility. They consider the construction of artificial life as one of the main scientific challenges of the synthetic biology era (cf. Tomita 2001).

## Science metaphors

Research areas such as molecular life sciences and genomics, one could argue, reflect the modern scientific tendency towards iconoclasm. Living beings are reduced to strands of letter-like symbols that can be analysed and manipulated on computer screens. At the same time, a plethora of images are produced by these sciences, and this includes the ubiquitous metaphor of the code: the tendency to refer to the logos-dimension of living beings (to the computational output of sequencing machines) as a “text” or a “code”: e.g. the “Morse code” of life (Schrödinger), the “barcode” of life (Strauss, 2009) or the “language” of life (Collins 2011). The genome as the “program” of life has become a ubiquitous metaphor, even a cliché. Indeed, it seems impossible to speak comprehensively about the output of genomics sequencing machines without the use (wittingly or unwittingly) of metaphors.

As indicated, metaphors are words, phrases or images that are applied to something to which they are not literally applicable, indicating some kind of analogy or similarity. It strikes me that, quite often, this involves the application of a term taken from the natural realm to something which rather belongs to the technological realm (i.e. things produced by us), and vice versa. For instance, if we compare a cathedral to a forest, or a forest to a cathedral, we are employing metaphors. A metaphor is not only a replacement (of one word by another), but also a *condensation* (“Verdichtung”, psychoanalytically speaking), in the sense that a number of key features are captured and brought together by the image. For example, if we call a forest a cathedral, we are emphasising features such as silence, darkness and the similarities between pillars and large trees. “Biobricks” (Hellsten & Nerlich 2011) and “building blocks of life” are likewise metaphors. A concrete, tangible human artefact (“brick”) is used to elucidate and condensate a complicated biological idea. The genetic “program” is obviously a metaphor, transferred from computer research into biology (from the technical into the natural, from in silico to in vivo). Considering the genome as the music score of life (Noble, 2008) is evidently metaphorical as well, and the same applies to the genome-as-a-map metaphor, employed during the press conference in June 2000, when the draft version of the human sequence was proudly presented to the world.

**Fig. 1:**
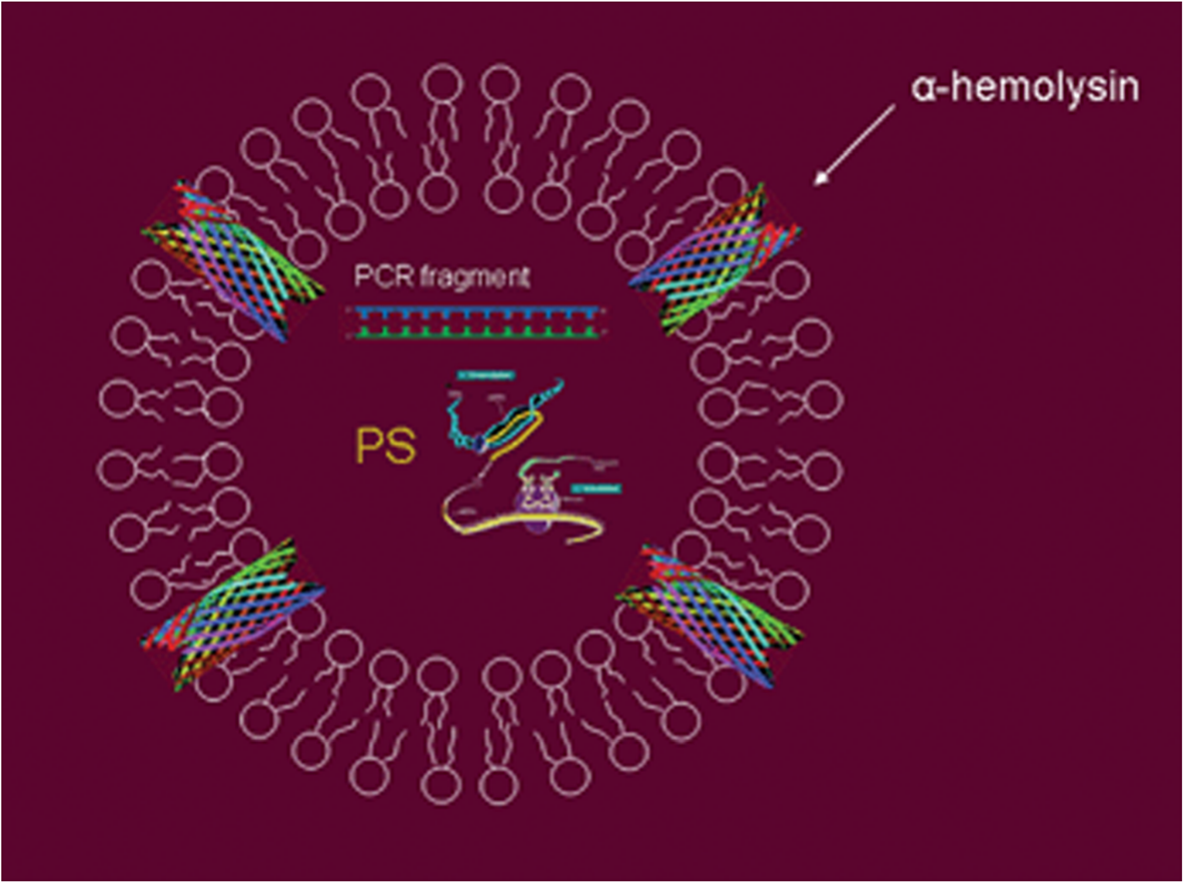
Artificial cell-based device

**Fig. 2:**
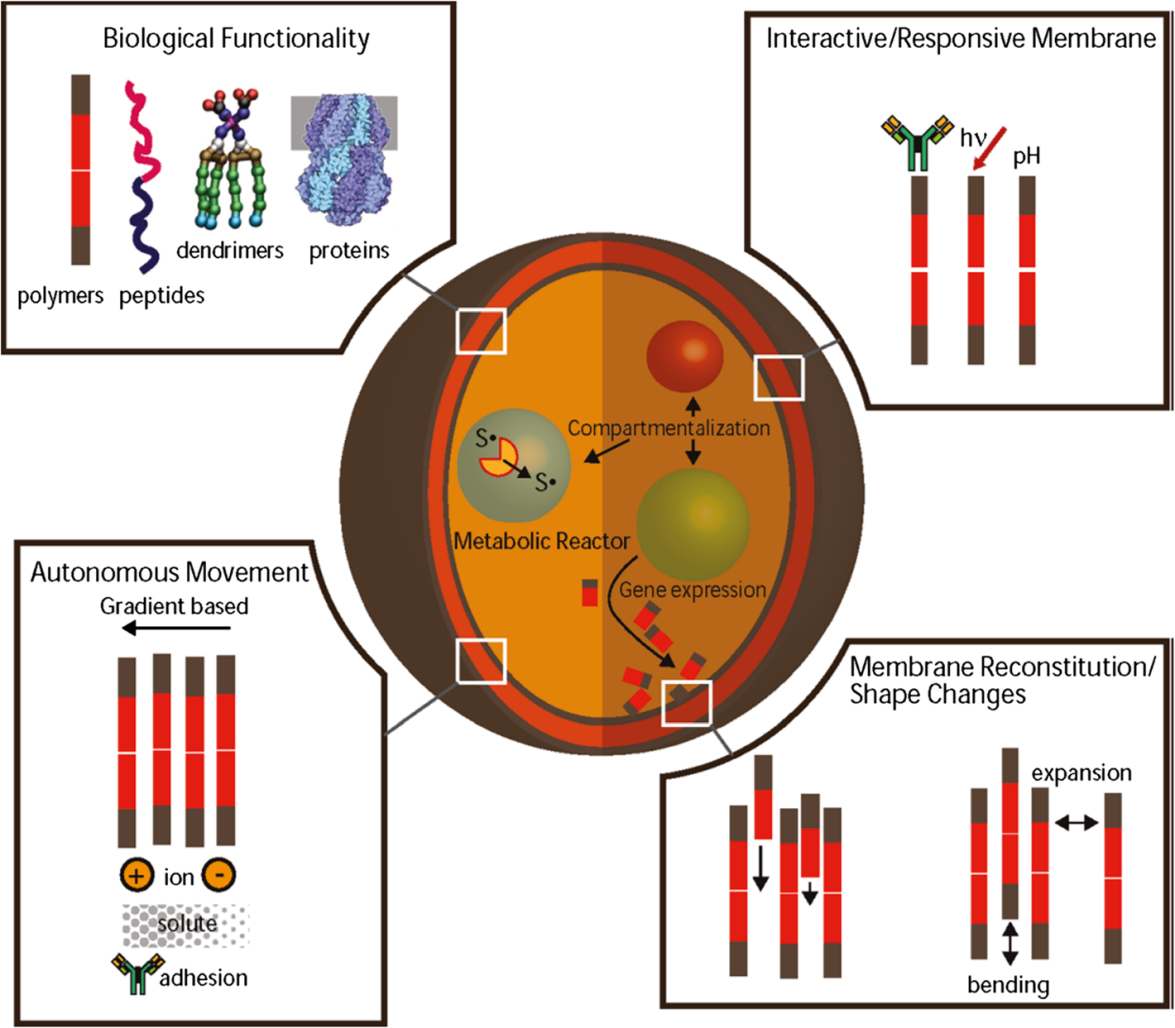
Protocell

**Fig. 3:**
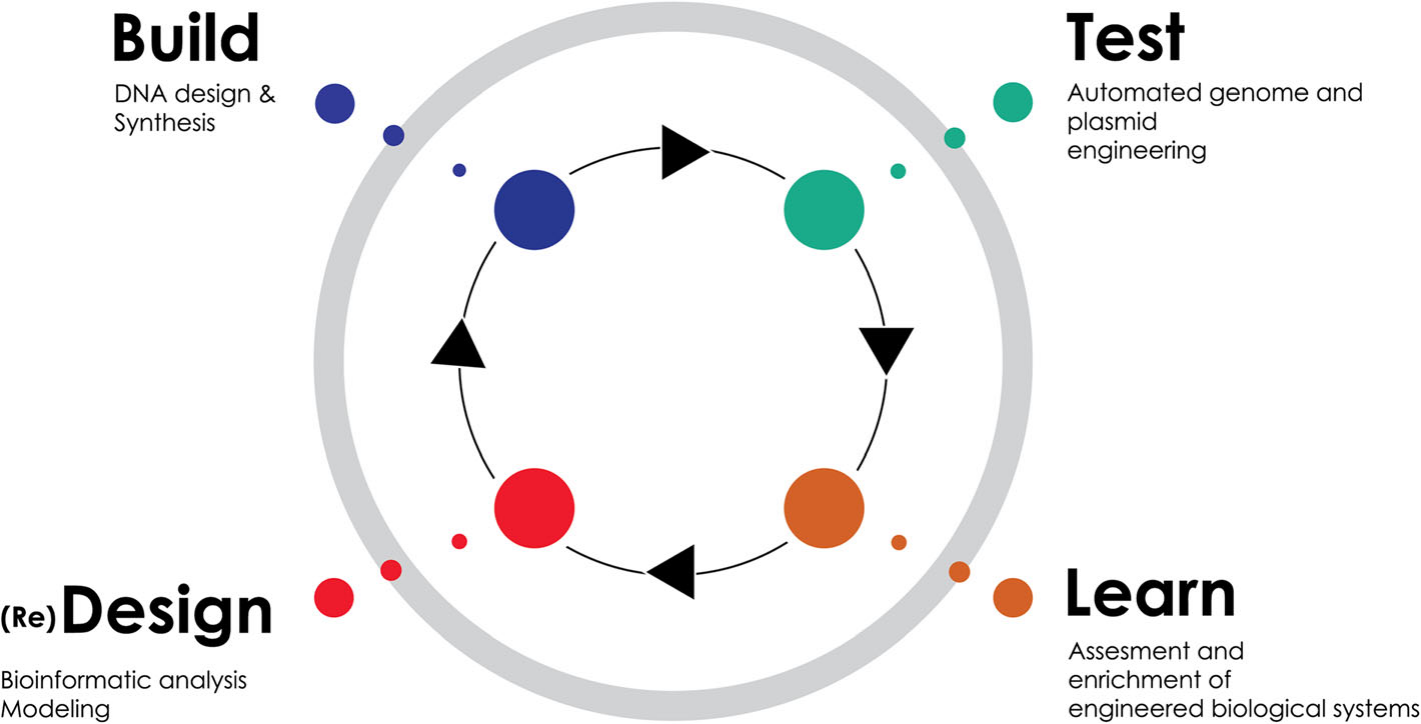
Synthetic Biology Foundry

Synthetic biology is a research field replete with metaphors (Hellsten & Nerlich 2011; McLeod & Nerlich 2017). The synthetic or artificial cell has been referred to as the Holy Grail of synthetic biology: a metaphor imported from alchemy and Christian mysticism. In this contribution, however, I want to consider one particular metaphor, namely the synthetic cell as a mandala. The mandala concept easily comes to mind whenever I see model versions of synthetic or artificial cells, inserted in academic papers or displayed on PowerPoint slides during meetings and conferences (Zwart 2018). Take for instance the following three examples of synthetic biological mandalas, more or less randomly selected from the literature. Figure 1 depicts an “artificial cell-based device” discussed by Giovanni Murtas (2009); Fig. 2 is a “protocell” meant to mimic a biological cell and discussed by Kamat et al. (2011); and Fig. 3 was found on the website of the Synthetic Biology foundry.^3^

Can such figures indeed be considered as mandalas? And if so, how does the mandala concept contribute to our understanding of synthetic biology in general and synthetic cell endeavours in particular? In the next section, building on the work of Carl Gustav Jung, I will explore the relevance of the mandala metaphor for current philosophical efforts to come to terms with synthetic biology as a converging research field.

## Life sciences mandalas

**Fig. 4:**
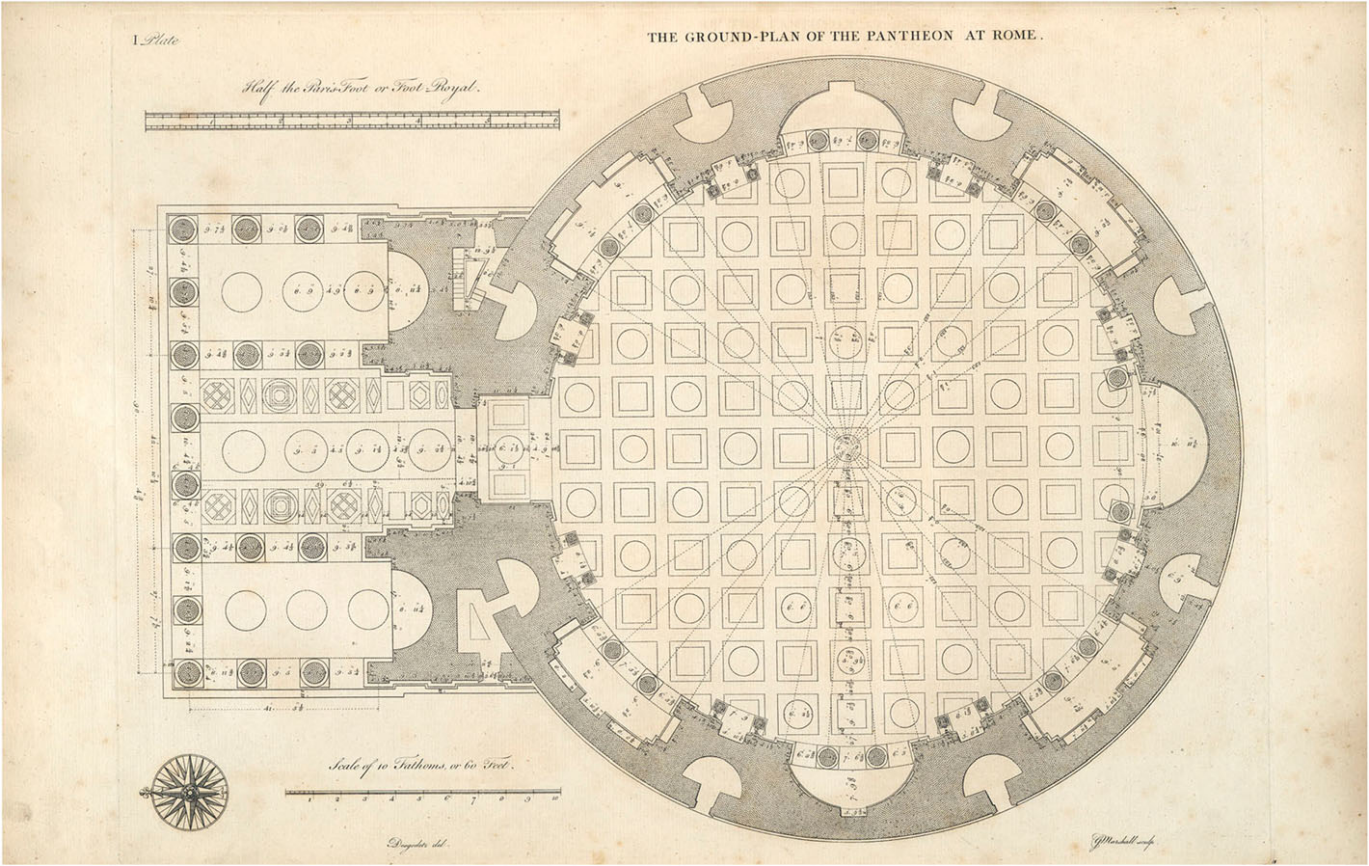
Ground plan Pantheon

A mandala (Sanskrit for circle or sacred circle), is a spherical-quadratic diagram. According to Jung, it is an archetypal symbol for unity or wholeness (Jung 1944/1968, p. 27; Jung 1950/1959, p. 356), a pattern of geometric shapes, contained within a circle or square (or “squared circle”), concentrically arranged and radiating from a centre. It is a harmonious, symmetric image that is gradually constructed, guided by active imagination (Jung 1944/1968, p. 96; Jung 1950/1959, p. 356). It contains everything and reveals how everything is related (Jung 1950/1959, p. 357). It may be the ground-plan for a building (a garden, a temple, a monastery courtyard, a city). The ground-plan for the Pantheon in Rome for instance can be considered a mandala: a spherical-quadratic building that contains everything (everything spiritual, as *pan-theon* means “all the gods”) (Fig. 4).

A mandala is often used as a “yantra” (literally: instrument or contraption): that is, as a visual aid in contemplative and meditative exercises (Jung 1950/1959, p. 356). But it may also function as a roadmap for processes of reconciliation and individuation. By realising wholeness, a mandala compensates for the contradictions, conflicts and disorderliness of actual reality (Jung 1944/1968, p. 27; Jung 1950/1959, p. 388). A mandala reflects and enables the transition from disorientation and confusion to order, balance and wholeness (Jung 1950/1959, p. 360). The centre has special symbolic relevance and may contain a symbol, a sacred text or a healing substance (φάρμακον). A mandala is a *coniunctio oppositorum* (a “union of opposites”) as Jung calls it, for instance: light and darkness, a circle and a square (cf. the Pantheon), as well as of the rational and the spiritual, the symbolical and the imaginary, etc. It is a symmetrical arrangement of seemingly disordered, contradictory and irreconcilable elements (Jung 1950/1959, 388). As the archetype of cosmic wholeness, it often reflects the shape of an eye or an egg. A mandala represents integration and homeostasis, but it is also the map or program for a long and difficult journey, with each layer representing a part of this journey (towards wholeness or individuation). The mandala is an “archetypal” or “cross-cultural” metaphor (Ceccarelli 2013), moreover, and Jung (1950/1959 and elsewhere) provides an extensive, comparative iconographic mandala anthology to substantiate his conviction that, although mandalas are quite prevalent in particular spiritual practices such as Tibetan Buddhism, they can in principle be encountered in all cultural traditions and historical periods.

What is the connection between mandalas and modern science? First of all, Jung commenced his systematic analysis of mandalas to understand the dreams of a famous scientist (a contemporary and acquaintance of Erwin Schrödinger in fact) who also was a prolific dreamer (one of the most famous dreamers in the history of psychoanalysis), namely Nobel laureate Wolfgang Pauli (Lindorff 1995, 2004). Pauli was a prominent quantum physicist who, among other things, postulated the existence of the neutrino in 1930 and acted as Mephistopheles in the famous Copenhagen version of Goethe’s Faust, written by Max Delbrück and performed in 1932 (Gamow 1966; Segre 2008). According to Jung, mandalas played an important role in Pauli’s dreamlife, perhaps to compensate for the disruptive impact of quantum physics on established worldviews.

**Fig. 5:**
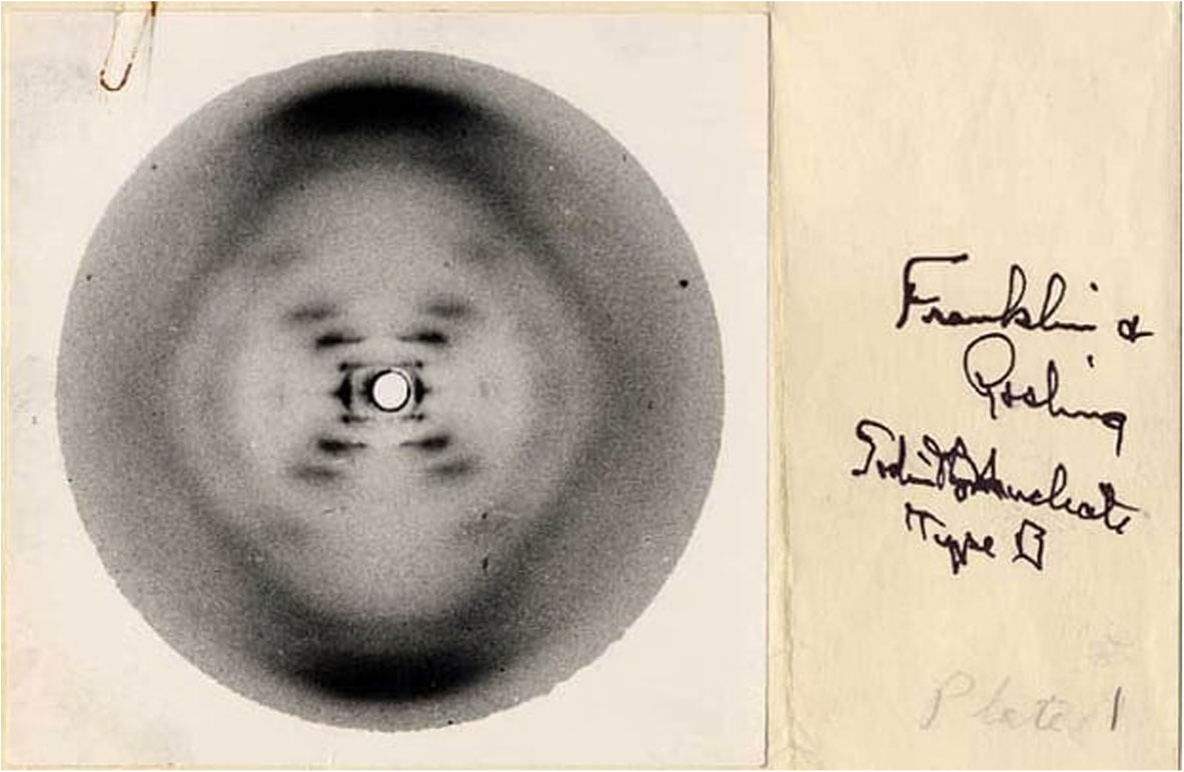
Photograph 51

But there are more mandalas showing up in modern science. One fascinating example is the famous *Photograph 51*, taken by Rosalind Franklin and her collaborator Raymond Gosling in 1952 and shown by Maurice Wilkins (without Franklin’s knowledge) to James Watson (in a corridor at King’s College, London) as a decisive piece of evidence for the helical structure of DNA. With the assistance of Wilkins, Watson was able to peep through the keyhole of Franklin’s laboratory, as it were: the primal science of molecular biology research, and a crucial step on the pathway that led to the discovery of DNA (Zwart 2015). This photograph (a helical structure, seen from above) reflects the archetypal structure of a mandala, which is no coincidence of course, for this picture is not only a ground-breaking effort to spectrographically capture what can be regarded as the essence of life, but also marks the commencement (*Anfang*) of a long and complicated project or journey, of which the synthetic cell would be the final completion (Fig. 5).

Archetypes are a priori mental forms or templates which realise themselves under certain circumstances in certain ways, but the general outline remains more or less constant. The mandala archetype (which can be found in all cultures and all periods of history) is considered by Jung as a symbol of restored unity or wholeness. And indeed, even in the technoscientific arena of synthetic biology research, mandalas can be found: in visualisations of biological structures, presented on PowerPoints during academic lectures or available on the Internet. Time and again, in journal articles and conference halls, the archetypal features of mandalas make their appearance.

But what can be the benefit of this exercise in pattern recognition? From the point of view of scientific iconoclasm, there may even be epistemic risks involved. As Conti et al. (2007) argue, for instance, scientists often try to create a semblance of order in the messy materials coming from their experiments by translating them into graph-like structures, with genes, protein, metabolites and their various interactions represented with the help of nodes and arrows. But although “scientists are in general very fond of these Mandala-like pictures” (Conti et al. 2007, p. 164), such visualisations may prove arbitrary and ad hoc, while the suggestion of completeness and transparency may actually be misguiding, for there is always *much more* to “chaordic” living systems^4^ than what is captured by such quasi-reassuring, intricate yet simplifying diagrams. Although (from the point of view of iconoclasm) caution, or even suspicion, concerning the use of mandala-like images is understandable, it does not answer the question *why* scientists involved in synthetic biology in general, and in the synthetic cell debate in particular, revert to producing such forms.

In the next section, I will shift the focus from general considerations (pertaining to synthetic biology discourse as such) to the individual or micro-epistemic level, using the mandala concept to analyse the active imagination of a group of early stage researchers invited to visualise the synthetic cell, in the form of a four-colour drawing.

## A philosophical experiment: active imagination and iconogenesis

How to study mandalas in contemporary synthetic biology discourse? Before discussing the results of the case study (the philosophy session) as such, allow me to briefly elucidate the methodology of a psychoanalytical approach to contemporary science. Rather than reflecting on molecules, molecular processes or synthetic cells, a psychoanalytical approach examines scientific research activities from an *oblique* perspective (Zwart 2017; cf. Babich 1994, p. 3), focussing on the interactions between scientific subjects and their objects (ranging from specific biomolecules up to organic or synthetic cells). At least three complementary strategies are available. First of all: discourse analysis, following the discursive flow (of academic papers, project descriptions or PowerPoint presentations produced by synthetic biologists) with *evenly-posed attention* (“gleichschwebende Aufmerksamkeit”) as Freud (1912/1943, 1917/1940, p. 297) once phrased it, focussing on certain concepts, terms or images that trigger the attention or catch the “philosophical ear” (Zwart 2017, p. 2). This method resulted in a first, exploratory analysis of three mandala-like illustrations (above), more or less randomly selected from the current synthetic biology literature.

A subsequent methodological option is to focus on a moment of commencement: a primal scene (*Urszene* or *Anfang*) of the research practice that eventually evolved into current synthetic biology discourse. An example of this approach is the discussion (above) of Rosalind Franklin’s crystallographic picture of DNA as a key-hole glance into the “essence” of life (“In the beginning, there was a photograph”).

**Fig. 6:**
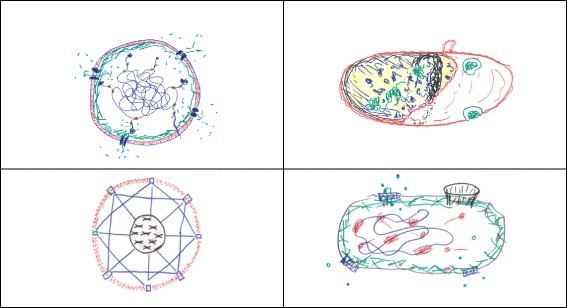
Synthetic cell drawings

A third and final option is to approach synthetic biologists, active in the field today, on the individual level. Rather than conducting interviews or participant observation, however, I opted for the Jungian technique of *active imagination*. During a philosophy session involving nine Ph.D. researchers enrolled in the synthetic cell project mentioned above, participants were invited to make a drawing of a synthetic cell. Below, a sample of four of these drawings is inserted. The session began with a short introductory lecture, but the mandala concept was not discussed until after the drawing assignment. I will now briefly analyse the results (Fig. 6).

What is remarkable, first of all, is that most of the drawings produced during the session are spherical. Following my conversations with senior researchers and principal investigators involved in the project, this need not be the case. For practical purposes, synthetic cells may well be cubical, or produced in dice-like formats. Most early-stage researchers involved in our session, however, envisioned the synthetic cell as a spherical entity (although one of the participants submitted a drawing of three spherical cells instead of one).

Most of the drawings, moreover, reflect a mandala-like shape (εἶδος). This notably applies to the two drawings depicted on the left. The bottom-right drawing can be regarded as “semi-mandala-like”, the overall shape being tubular or elliptic rather than spherical, but the upper-right drawing is definitely a *non-mandala* image. In their comments, the participants who produced Mandala-like drawings indicated that this shape for them expresses aspects of synthetic cells such as “equilibrium”, “balance”, “homeostasis”, “rational design” and “biomimesis”. On the other hand, the participant who produced the non-Mandala drawing commented that, for her, synthetic cells represent “artificiality”. More specifically, her drawing was meant to reflect the abundant “fullness” of cells, either artificial or living, compared to the empty cells spotted by Robert Hooke 1665.

One of the striking features of mandala-like cells depicted above, I would argue, is the number and position of the orifices (or “gates”, as Jung would call such features). In the selected drawings, four (on two occasions) or even eight (on one occasion) orifices (or gates) are located in a spherical membrane. The upper-left drawing is strikingly symmetrical, with evenly distributed orifices and a “symbolic” core (containing genetic information: the cell’s “program” or “sacred text”, located in the centre). In the lower-left mandala, the orifices are equally (symmetrically) distributed once again, but the genetic (symbolic) information is now wrapped in a second (nuclear) membrane. Another striking feature of the lower-left mandala is the straight and diagonal connecting lines between the gates, reminiscent of a Buckminster Fuller-like pattern or a La Plata street map. The drawing at the bottom-right side is semi-Mandala-like: skewed or elliptical rather than spherical, with four orifices or gates, one of which is more pronounced (reflecting what Jung would refer to as the quaternity ratio, 3:1). The biggest opening seems a kind of fistula, moreover, apparently created there on purpose, allowing for chemical substances to be administered. The non-mandala drawing (upper-right) reminds me of an egg-shaped organism, with a gut-like area on the left and two green features that look like eyes. Overall, without making any *quantitative* empirical claims (given the limited sample size), I would argue that (notwithstanding the various differences between these drawings), the mandala structure is a noteworthy feature.

From a Jungian perspective this is not only understandable, but also quite significant. As indicated, a mandala is an archetypal symbol of wholeness, and the synthetic cell can be regarded as a bio-molecular microcosm, a structure that contains and assembles “everything”: everything currently known about the biochemical structures and processes of cellular life, while allowing us to discern how all these processes and components are interconnected, with the cell membrane as the protective circle and the cell nucleus as the centre. Following Jung, besides representing the basic ground-plan or architecture of a synthetic cell, the mandala-like structure may also be regarded as a visual aid or roadmap for the process of building such a cell. In the course of the twentieth century, living cells were broken down into elementary molecular components as we have seen, representable with the help of alphabets of bio-scientific “characters” (symbols): the basic building blocks of life (nucleotides, genes, amino acids, proteins, etc.). The objective of building a synthetic cell is to bring all these components together again. Therefore, synthetic biology is often regarded as “holistic”, even by authors for whom the “esoteric” connotation of the term causes unease (Conti et al. 2007, p. 161).

**Fig. 7:**
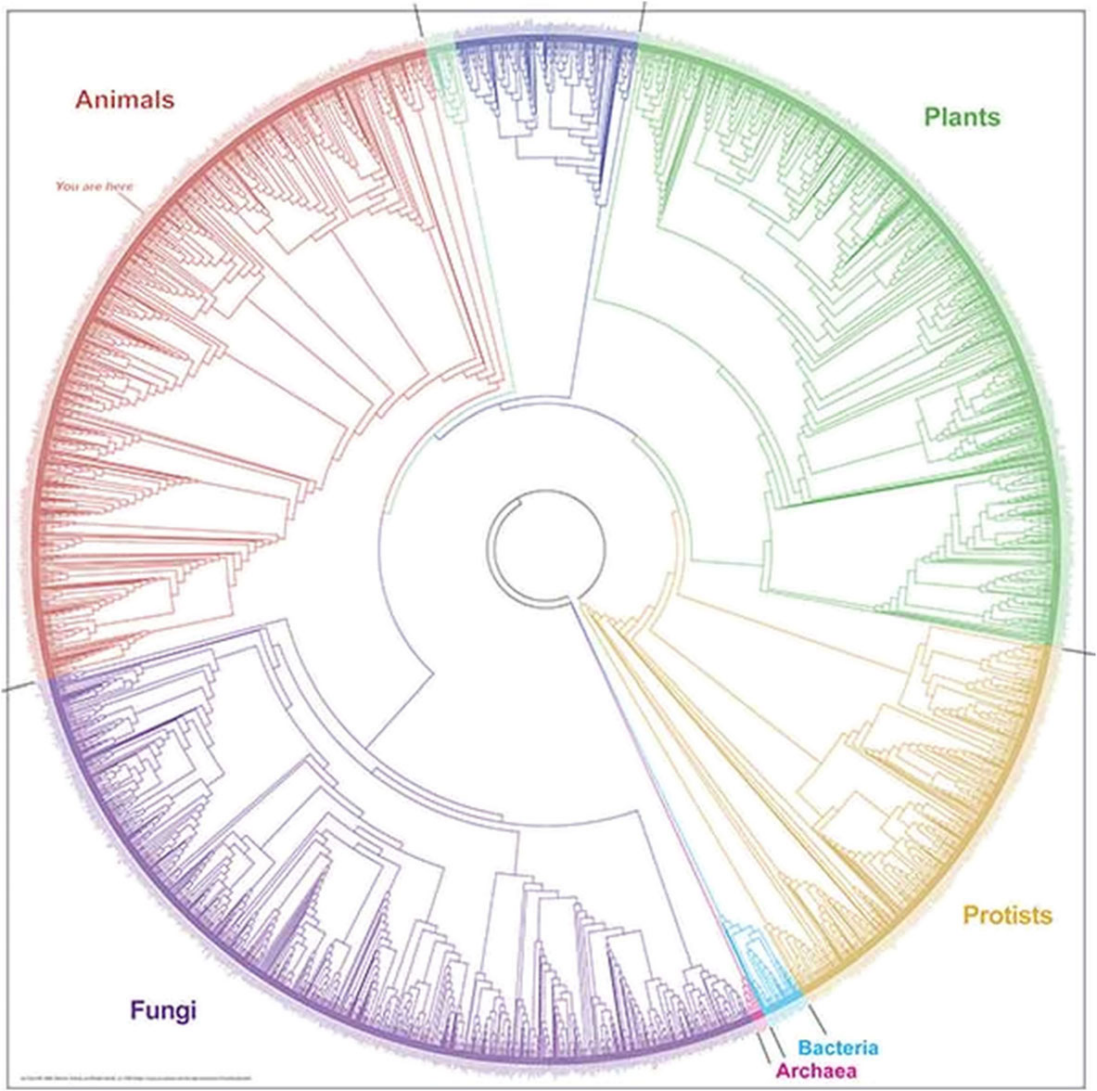
Tree of life

Mandalas can be encountered in other research fields as well; it is not an exclusive feature of cell synthesis. In phylogenetic research, for instance (studying the evolutionary history of and relationships among organisms), “phylogeny mandalas” (Hasegawa 2017) are used to visualise the Tree of Life. An impressive example is the version available on the *Global Genome Initiative* portal, inserted below (http://ggi.eol.org/about). Here again, I would argue that this image reflects the objective to reassemble scattered genomics information into a comprehensive whole, a phylogenetic pantheon if you like, in order to assess what we have learned during the obliteration stage (Fig. 7).

In psychodynamic terms, the building of a synthetic cell may be regarded as a collective exercise in reparation. All the “partial objects” of life sciences research (chromosomes, ribosomes, membrane, lipids, and so on) are allocated a functional place within the synthetic cell as a holistic, all-encompassing, pantheon-like assembly. As a model or structure, it is an imaginative condensation of molecular biological knowledge, and the synthetic cell emerges gradually, step by step, through active imagination. The centre consists of the nucleus containing the program (λόγος) of the cell, the core concept which realises itself in the visible, functioning structure. Mandala-like representations mimic the basic (circular or egg-like) form (εἶδος) of a biological cell, while realising the program (λόγος) as well. Although a synthetic cell may not be an exact replica of a living biological cell (probably it will be a highly simplified version), it presents the general outline or model: the overall idea (εἶδος). This explains why most of the synthetic cells envisioned by the participants in the session described above mimic and realise the archetypal, spherical structure of a biological cell, exemplifying balance and homeostasis: the equilibrium of multiple counteracting and apparently incompatible forces (*coniunctio oppositorum*). A synthetic cell diagram reflects the archetypal form (εἶδος) of a mandala and may serve as a visual aid allowing researchers to envision and synthesise the various biochemical, biomolecular and bio-computational fragments into a comprehensive whole. Thus, the mandala represents the resurgence of the form (εἶδος), or rather: the synthesis (or reconciliation) of form (εἶδος) and formula (λόγος), but now in vitro.

## Concluding remarks: responsible metaphor management

Still, this is only part of the story, for the correspondences between synthetic cell diagrams and mandalas not only pertain to the object pole (the end product of the synthetic cell endeavour), but also to the subject pole (the researchers of research teams involved, Zwart 2017). As an analytical psychologist and psychotherapist, Jung was interested in the scientific subject, more than in the object. As a concretisation of the mandala archetype, the synthetic cell concept plays a role in the process of individuation, so that synthetic biology research becomes a practice of the Self. Whereas in the past researchers and research teams were working under the sway of specialisation, focussing on partial objects (on very specific molecules or molecular processes), the synthetic cell (reflecting the holistic turn in contemporary life sciences research) allows for convergence, also in terms of the research programs and research activities involved. After decades of reductionism and fragmentation, researchers are now again envisioning the cell *as a whole*. It is only by reconstructing this microcosmic whole that a cell can be truly understood. The cell is a microcosm, a condensation of living nature as such, and the synthetic or holistic turn in biology concurs with a process of integration on the part of the scientific subjects themselves.

Synthetic cell projects such as BaSyC assemble experts representing multiple disciplines and approaches, so that the synthetic cell becomes a kind of scientific *Gesamtkunstwerk* (total work of art), actively engaging a significant sample of contemporary technosciences. Schrödinger’s argument that, in order to elucidate the enigma of life, biologists and physicists should learn to collaborate still conveys the basic logic of such a program. The disruptive impact of elementary particle physics and elementary particle biology (i.e., molecular life sciences research) is now assembled into the synthetic cell as closure. The synthetic cell mandala suggests that, within this closure, all parts and processes are interconnected, but it may also represent a map guiding the researchers involved on their journey to new insights, turning research indo self-edification, so that the building of a synthetic cell entails an element of Self-*Bildung* as well.

From a Jungian perspective, moreover, synthetic cell mandalas must be seen as performative compensations for current deficits. In terms of diagnostics, the present state of research in synthetic biology is “chaordic” (blending characteristics of chaos and order). In an imaginative manner, mandalas represent a complementary moment of compensation, counteracting the iconoclastic tendencies of technoscience towards quantification, computation and datafication. Dialectically speaking: the initial whole (the living organism: the first moment) is *negated* (obliterated into data: the second moment), but this inevitably results in the sense that we have lost something (the living cell, the organism as such), from which arises the urge to recover a more comprehensive, holistic view (the *negation of the negation*: the third moment), to which active imagination can contribute (as a form of reparation). The role of philosophy is not only to highlight and analyse, but also to foster such a dialectical dynamics. Whereas technoscientific laboratories create optimal conditions for reductionism and iconoclastic obliteration (“negation”, the second moment), training sessions such as the one described above may be regarded as philosophical laboratories, creating optimal conditions for fostering the resurgence of the (third) holistic moment. In his final publication (his testament as it were, again building on Jung), Gaston Bachelard (1960) thematised this relationship between *iconoclasm* (abstract, quantitative thinking) and *imagination* (reverie) in terms of *animus* and *anima*, arguing that, in order to overcome epistemic paralysis, we need mutual exposure between the two. Therefore, we need to invest in (and critically consider) both dimensions: iconoclasm and imagination.

The mandala signifies an ideal end state of convergence. Yet, while the synthetic cell mandala symbolises the inviting future, in real life researchers remain challenged by multiple conflicts, tensions and frustrations (see for instance McLeod et al., 2017). The synthetic cell mandala may work as a psychic aid, guiding researchers in their efforts to face the realities of laboratory life and its adverse impacts. As a visual representation of a common goal or ideal, life sciences mandalas (presented in bright colours during lectures for instance) may have a performative effect, fostering team building and acting as a source of inspiration. Rather than seeing synthetic cells as reifications of an archetypal symbol, we conclude that the synthetic cell mandala functions as a regulative idea, expressing a sense of direction, oriented towards living systems as organic wholes. It represents a sublated and reflected use of metaphors, which has been referred to as “responsible metaphor management” (Verbrugge et al. 2016).

## Abbreviations

BaSyC: Building a synthetic cell project

## Footnotes

1
https://www.nwo.nl/en/research-and-results/research-projects/i/45/29045.html

2 Cf. “Whereas the comparative anatomy or morphology of animals and plants, based on collection, observation, comparison, and description, was the definitive technique for the classification of life forms during the classical period of natural history, it is molecular biology that today provides the primary analytic perspective on the essence of life and its defining mechanisms… What is considered to be “the stuff of life” in modern scientific terms [i.e. DNA, composed of nucleotide chains that guide the manufacture of essential proteins, that all living beings are now known to have in common] is today more similar to biochemistry than to zoology” (Franklin 1995/2014, p. 1811).

3
http://syncti.org/research/synthetic-biology-foundry/

4 The portmanteau term indicates that they are mixtures of chaos and order (Hock 1999; Conti et al. 2007).
